# Effects of vision impairment on cognitive function: the bidirectional chain mediating role of sleep quality and psychological disorders

**DOI:** 10.3389/fpubh.2025.1611723

**Published:** 2025-10-01

**Authors:** Minghao Yu, Hangqing Zhou, Yi Zhao, Hanna Lu, Yan Shao

**Affiliations:** ^1^Tianjin Key Laboratory of Retinal Functions and Diseases, Tianjin Branch of National Clinical Research Center for Ocular Disease, Eye Institute and School of Optometry, Tianjin Medical University Eye Hospital, Tianjin, China; ^2^School of Basic Medical Sciences, Tianjin Medical University, Tianjin, China; ^3^Department of Psychiatry, The Chinese University of Hong Kong, Shatin, Hong Kong SAR, China

**Keywords:** Health and Retirement Study, sleep quality, psychological disorders, vision impairment (VI), cognitive function, chain mediation model

## Abstract

**Introduction:**

Vision impairment (VI) and cognitive function have profound impacts on quality of life, but there is still a lack of comprehensive research on the connection between VI and cognitive function. The study was designed to investigate factors influencing cognitive function, analyze the link between VI and cognitive function, and explore how sleep quality and psychological disorders mediate this relationship.

**Methods:**

The study utilizes survey data from the Health and Retirement Study (HRS) involving 10,884 older adults aged 50 and above in the United States. Pearson correlation analysis was conducted to elucidate the associations between the variables, and the R package bruceR (version 2024.6) was used to analyze multiple mediation effects through model 6.

**Results:**

VI is positively correlated with sleep quality and psychological disorders, and negatively correlated with cognitive function. Sleep quality is positively correlated with psychological disorders and negatively correlated with cognitive function. Psychological disorders are negatively correlated with cognitive function. All correlations are statistically significant. VI directly impacts cognitive function while also indirectly influencing it through sleep quality, psychological disorders, and the bidirectional mediating chain connecting these factors.

**Conclusion:**

Sleep quality, psychological disorders, and their bidirectional relationships mediate the effect of VI on cognitive function in aging populations. Through this study, we gain a more profound comprehension of how VI relates to cognitive function. In the future, cognitive enhancement in individuals with VI could be achieved by improving sleep quality, addressing psychological disorders, or integrating assessments of these factors into the evaluation of cognitive function.

## Introduction

With the aging of society, age-related cognitive decline is becoming a health issue of global concern. According to the World Health Organization (WHO), 684,000 people die every year due to falls, most of which occur in the older adult population aged 60 and above, with cognitive decline being one of the major risk factors (1). An individual's cognitive function encompasses multiple dimensions, such as attention, thinking, memory, executive function, and expressive language skills (2). Intact cognition is the fundamental capacity for people being able to carry out their daily activities, work, and social interactions. To accomplish this, Schubert et al. (3) conducted a series of tests to assess an individual's cognitive function by whether or not they can complete the test and how long it takes to complete the test. Under the influence of various factors such as aging and illness, individuals inevitably experience a decline in cognitive function, which may include, but are not limited to, slowed thinking, impaired language expression, difficulty in concentrating, impaired judgment, and memory loss (4). Cognitive decline, not only reduces the wellbeing and satisfaction of daily life at an individual level but also increases the burden in terms of family caregiving and socio-economic (5). In response to the controversies on the mechanisms of age-related cognitive decline, studies from different research fields have provided evidence of possible triggers for cognitive decline. For example, a study from 2020 showed that aging, gender, and family history appeared to be immutable risk factors for cognitive function, while nine modifiable factors, such as living environment, years of education, living alone, and marital status, could be better modified to improve an individual's cognitive function (6). Based on the individual's sleep process featured by electroencephalogram (EEG), Tarokh et al. (7) introduced the critical roles of sleep neurophysiology in the studies of cognitive function and obtained a series of neurophysiological data by measuring sleep EEG profiles through nighttime recordings, which led to the hypothesis of a key correlation between the level of cognitive performance and the development of the sleep spindles in adolescents (8). Similarly, another study recruited older adults aged 65 and over and observed significant associations between vision impairment (VI), mixed sensory impairments, and severe cognitive impairments, however, a similarly significant association was not found for auditory impairments (9). Thus, despite the extensive body of research conducted across numerous domains, the risk factors and underlying mechanisms contributing to cognitive decline remain insufficiently understood and warrant further in-depth investigation.

It is impossible to overlook the fact that the global vision landscape has been steadily deteriorating over the past decade, largely due to the compounding effects of population growth and aging. According to WHO estimates, by 2050, there will be a substantial rise in the global prevalence of myopia and high myopia, impacting nearly five billion and one billion individuals, respectively. Additionally, an estimated 500 million individuals will endure uncorrected near vision disorders, primarily due to the lack of access to reading glasses (10). As outlined by the American Academy of Ophthalmology, objectively measured VI encompasses impairments in distance vision, near vision, and contrast sensitivity. In contrast, self-reported VI relies on an evaluation by the individual's subjective report, or their representative, regarding their visual acuity. This assessment includes various aspects, such as whether the individual is already blind or experiences difficulties with distance or near vision without corrective lenses (11). A previous report posited that enhancing human eye health should be prioritized as a critical aspect of human development. It highlighted that VI already permeates and impacts various domains, including body health, mental health, cognitive function, social tasks, and quality of life. The report also underscored a preliminary correlation between VI and cognitive function, though the reliability of this relationship requires further exploration through additional studies (12). However, in Hreha's et al. (13) study, the findings contradicted previous assumptions, indicating that VI had no relationship with the rate of cognitive decline in stroke patients. As such, the implications of VI for cognitive function remain uncertain. By proactively investigating the mechanisms through which VI could influence domain-specific cognitive function, we can enhance our understanding of VI and cognitive function while also formulating strategies to boost the quality of life for older individuals.

Sleep stands as one of the most universal and essential human activities, playing a pivotal role in sustaining the body's normal functions. Good sleep quality is the cornerstone of older people's vitality, and it is particularly important for their brain health and wellbeing. Research has shown that individuals who consistently enjoy sufficient sleep exhibit lower risks of various adverse health conditions and perform better in tasks requiring information integration and emotional regulation compared to those who are suffering from sleep-deprivation (14). Accordingly, it is widely believed that sleep disturbances are linked to various health problems such as stress, depression, suicidal thoughts, psychiatric disorders, chronic disease risk, and mental decline, and the more severe the sleep disorder, the more difficult it is for subjects to concentrate and make accurate judgments, as evidenced by the research of sleep disturbances on cognitive function (15, 16). One study indicates that individuals with VI often experience poor sleep quality compared to their sighted counterparts, suggesting a potential association between VI and sleep quality that warrants further investigation (17). Inspired by this, in an aging population, we hypothesized that sleep quality may act as a mediator linking VI and cognitive function.

Mental health is defined as a condition of positive wellbeing where people can fulfill their potential, experience joy, navigate stress, sustain meaningful relationships, and work productively. It profoundly shapes how people perceive their emotions, process thoughts, and conduct their lives (18). Declining mental health has emerged as a global concern because it impairs social function and cognitive performance. Research indicates that individuals with VI are often more susceptible to anxiety and depression due to factors such as social isolation, adjustment challenges, and future uncertainties. Compared to their peers with normal vision, those with VI may exhibit a poorer psychological state (19–21). A research focusing on aging populations in Amsterdam found the linkage between depressive symptoms and cognitive decline. The findings revealed that depressive symptoms could significantly hinder the speed of information processing in older patients. Additionally, individuals with impaired cognitive function were more susceptible to psychiatric disorders, highlighting the intricate interplay between mental health and cognitive performance in aging populations (22). Interestingly, some studies have suggested that VI can negatively affect social network size, which in turn leads to shrinking social circles and social isolation (23–25). Research on how social isolation relates to psychological disorders is already well-founded, which at the same time provides evidence for an association between VI and psychological disorders (26). Based on the aforementioned studies, it is evident that there exists a complex interplay between VI, psychological disorders, and cognitive function. This leads us to hypothesize that one of the mediators in the relationship between VI and cognitive function in aging populations is psychological disorders.

**Figure 1:**
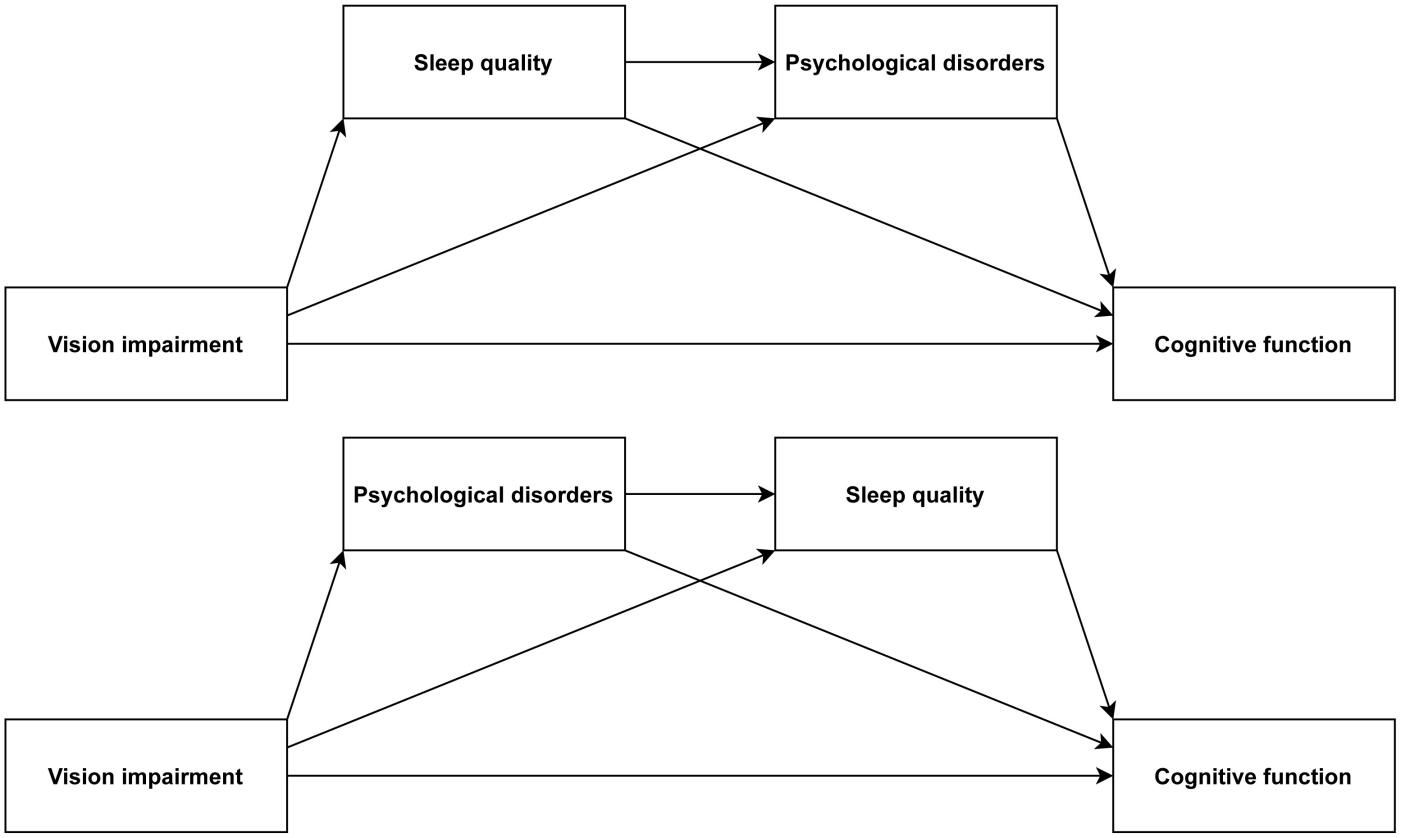
Chain mediation model.

Prior research has identified correlations between VI, sleep quality, psychological disorders, and cognitive function. Nevertheless, there is still a shortage of sufficient evidence at present, requiring additional efforts for in-depth investigation of the relationships and potential pathways of these variables. Thus, this study would focus on exploring the associations among these factors in an aging population, especially emphasizing the chain mediation roles that sleep quality and psychological disorders play in the interplay between VI and cognitive function. The hypothesized model is presented in Figure 1.

## Method

### Data sources and sample

As a continuous national longitudinal survey, the Health and Retirement Study (HRS) surveys Americans who are 50 years old and older, along with their spouses, irrespective of the spouses' ages. The research, funded by the National Institute on Aging (NIA U01AG009740), is carried out by the University of Michigan and involves surveying about 20,000 community-dwelling individuals. The core HRS survey commenced in 1992–1993 (wave 1) and follows up every 2 years, gathering information on various demographic factors, health events, and financial aspects from the participants. Comprehensive details regarding the HRS core survey questions, sampling methodology, and sample characteristics are documented in the earlier study (27). The data used in this study was approved by the institutional review board. Each participant signed an informed consent form. Additionally, the Human Research Protection Program at UCSF exempted the need for further consent from patients for research involving this existing dataset.

In this study, we utilized the research data from HRS 2018–2020, using the 2018 survey as the baseline and collecting cognitive function data from 2020 as the outcome variable. From the baseline, we initially had 17,146 participants. We excluded individuals under the age of 50 (*n* = 454), those with missing baseline information (*n* = 3,666), those lost to follow-up (*n* = 1,963), and those with missing outcome variables (*n* = 179). Ultimately, 10,884 older adults met the criteria for analysis.

## Measurements

### Vision impairment

During the primary interview, participants were questioned: “How would you rate your eyesight and near/distal vision using glasses or corrective lenses?” Their responses were evaluated on a five-level scale ranging from excellent to poor. Vision impairment classification is applied to individuals describing their vision as fair or poor, even with the use of glasses (28).

### Cognitive function

By using a modified Telephone Interview Cognitive Screen (TICS-m), the researchers were able to better assess participants' cognitive function (29). The HRS TICS-m was designed to evaluate various cognitive aspects, such as language-based memory, orientation, executive function, and attention. This instrument includes: (a) immediate and delayed word recall with scores ranging from 0 to 20; (b) a serial seven subtraction task, scored from 0 to 5; and (c) a backward counting task, with scores ranging from 0 to 2. The TICS-m was given to every participant at each wave of the study. It is frequently utilized to evaluate overall cognitive function (29, 30) and is recognized as a dependable and valid tool for telephone assessments aimed at detecting dementia (29). A composite score was derived from all the items to represent cognitive function, ranging from 0 to 27, with higher scores suggesting superior cognitive performance. The TICS-m scores from the years 2018 and 2020 were utilized for this study.

### Sleep quality

Sleep quality was assessed through five components, with four components scored on a one to three Likert scale and one component recorded as a binary variable. At each wave, participants reported symptoms of insomnia. The questions gauged issues related to falling asleep, maintaining sleep, waking up too early, and non-restorative sleep. Participants reported how often they experienced challenges such as “waking up in the middle of the night,” “getting up too early to return to sleep,” and “difficulty falling asleep,” as well as how frequently they felt “well-rested” upon waking in the morning. The response choices provided for these questions were “most of the time,” “sometimes,” and “rarely or never.” Furthermore, participants were asked if they regularly used medication to assist with sleep. The scoring for the items was reversed to accurately reflect that higher scores correspond to poorer sleep quality (1 = good quality, 2 = medium quality, 3 = poor quality), and the medication usage was scored separately (0 = no, 1 = yes). Finally, the five components were summed and adjusted to create a new scale that ranges from 0 to 9 (31).

### Psychological disorders

The mental health status was evaluated through the eight-item short form of the Center for Epidemiologic Studies Depression Scale (CES-D-8) (32). This eight-item scale serves as a significant tool for evaluating depression. Referring to a prior study (33), we opted to use the term “psychological disorders” in place of “depression.” The CES-D scale evaluated how often certain emotions occurred over the past week through eight dichotomous items, which included “happy,” “life was enjoyable,” “depressed,” “sad,” “unable to get going,” and “everything was an effort” (34). The scoring for the items “life was enjoyable” and “happy” was reversed before computing the total. These are total scores between 0 and 8, with higher values representing superior severity of psychological disorders.

### Control variables

We used information from the 2018 participants as control variables. The variables taken into account included: age, gender (male, female), educational levels (less than upper secondary, upper secondary and vocat, tertiary), marital status (married, single or other), total wealth (lowest quartile, Quartile 2, Quartile 3, highest quartile), drinking status (non-drinker, drinker), smoking status (never, current, former), BMI (underweight, normal, overweight, obesity), residence (urban, rural), number of diseases (0, 1, ≥2).

The categories of marital status include “married,” “married, spouse absent,” “partnered,” “separated,” “divorced,” “widowed,” “never married,” and “separated/divorced.” We grouped the options “married” and “married, spouse absent” under the category “married,” while other categories with smaller sample sizes were grouped as “single or other.” The total wealth (encompassing housing, vehicles, savings accounts, and more) minus any outstanding debts has been evaluated (including secondary residences, if applicable) in local currency (Dollars) and subsequently categorized into four groups according to quartile ranges (35). Individuals' smoking status and drinking behavior were defined based on their self-reported information concerning any prior use of alcohol or tobacco. The statuses of BMI are divided into four categories based on WHO guidelines: underweight (< 18.5 kg/m^2^), normal (18.5–24.9 kg/m^2^), overweight (25.0–29.9 kg/m^2^), and obesity (≥30.0 kg/m^2^). The types of diseases include: (1) hypertension (high blood pressure); (2) diabetes mellitus (elevated blood sugar levels); (3) cancer or any malignant tumor, excluding skin cancer; (4) chronic lung conditions other than asthma, such as emphysema or chronic bronchitis; (5) heart-related issues, including heart attack, coronary heart disease, angina, heart failure, or other cardiac problems; (6) stroke or transient ischemic attack (TIA); and (7) arthritis or rheumatism.

### Statistical analysis

Descriptive statistics analyzed the demographic characteristics, and Pearson's correlation analysis evaluated the relationships among VI, sleep quality, cognitive function, and psychological disorders. The representation of continuous variables involved means and standard deviations (SDs), while categorical variables were shown as counts along with their relevant percentages. After grouping participants according to the presence of VI, differences in categorical variables were evaluated using Pearson's Chi-squared test, while the Wilcoxon rank-sum test was applied to analyze differences in continuous variables. Furthermore, the R package bruceR (version 2024.6) (36) was utilized to investigate how VI influences cognitive function via the mediation of sleep quality and psychological disorders. In this study, 5,000 bootstrap samples were applied to establish the 95% bootstrap confidence interval (CI). For a mediating effect to be regarded as significant, the 95% confidence interval should not include 0. All statistical tests employed were two-tailed, and significance was determined at a threshold of *P* < 0.05. Statistical analysis was conducted using R 4.4.1 (R Core Team, Vienna, Austria).

## Result

### Characteristics of the participants

Table 1 contains a summary of the study participants' details. Among the 10,884 individuals, 3,314 (30.4%) were identified with VI, while 7,570 (69.6%) were free from VI. Those with VI have an average age of 66.6, with 61% being female; 61% having a secondary education level, and 39% having total wealth in the first quartile. Non-Hispanic whites constitute 50%, with non-smokers and former smokers each accounting for 41%. Approximately half are drinkers, and about half are married, while 74% have two or more chronic diseases, 76% live in rural areas, and a higher proportion have an elevated BMI. For those without VI, the average age is slightly older at 67.0. A higher proportion, 34%, have tertiary education, and 58% have total wealth in the top two quartiles. Non-Hispanic whites also make up the largest proportion at 69%. Non-smokers are slightly more prevalent, accounting for 49%, while drinkers are more common at 62%. A larger proportion are married, at 61%, and those with two or more chronic diseases constitute a smaller percentage at 61%. Similarly, 76% live in rural areas, and the majority also have a higher BMI. Cognitive function declined over time in both groups, but the cognitive function of the VI group was significantly lower than that of the non-VI group (2018: 14.6 vs. 16.6; 2020: 14.1 vs. 16.3). Similarly, the VI group exhibited poorer mental health (2.2 vs. 1.0) and sleep quality (3.7 vs. 2.5) compared to the non-VI group.

**Table 1:** Descriptive statistics of baseline information in 2018.

**Variables**	**Non-VI**	**VI**	***P*-value^b^**
	***N* = 7,570^a^**	***N* = 3,314^a^**	
Age	67.022 (10.013)	66.623 (10.396)	0.009
**Gender**			
Male	3,156 (42%)	1,285 (39%)	0.004
Female	4,414 (58%)	2,029 (61%)	
**Education**			
Less than upper secondary	550 (7.3%)	748 (23%)	< 0.001
Upper secondary and vocat	4,423 (58%)	2,019 (61%)	
Tertiary	2,597 (34%)	547 (17%)	
**Total wealth**			
Q1	1,441 (19%)	1,294 (39%)	< 0.001
Q2	1,775 (23%)	933 (28%)	
Q3	2,099 (28%)	621 (19%)	
Q4	2,255 (30%)	466 (14%)	
**Race**			
Hispanic	477 (6.3%)	502 (15%)	< 0.001
Non-Hispanic Black	1,518 (20%)	990 (30%)	
Non-Hispanic White	5,240 (69%)	1,642 (50%)	
Other	335 (4.4%)	180 (5.4%)	
**Smoking status**			
Never	3,738 (49%)	1,351 (41%)	< 0.001
Current	750 (9.9%)	609 (18%)	
Former	3,082 (41%)	1,354 (41%)	
**Drinking status**			
Non-Drinker	2,866 (38%)	1,609 (49%)	< 0.001
Drinker	4,704 (62%)	1,705 (51%)	
**Marital status**			
Married	4,600 (61%)	1,606 (48%)	< 0.001
Single or other	2,970 (39%)	1,708 (52%)	
**Number of diseases**			
0	1,122 (15%)	290 (8.8%)	< 0.001
1	1,850 (24%)	561 (17%)	
≥2	4,598 (61%)	2,463 (74%)	
**Residence**			
Urban	5,725 (76%)	2,513 (76%)	0.8
Rural	1,845 (24%)	801 (24%)	
**BMI**			
Normal	1,863 (25%)	743 (22%)	< 0.001
Obese	2,759 (36%)	1,372 (41%)	
Overweight	2,859 (38%)	1,145 (35%)	
Underweight	89 (1.2%)	54 (1.6%)	
Cognitive function (2018)	16.624 (4.023)	14.556 (4.367)	< 0.001
Cognitive function (2020)	16.274 (4.424)	14.089 (4.850)	< 0.001
CES-D score	1.023 (1.621)	2.158 (2.293)	< 0.001
Sleep quality	2.522 (2.089)	3.692 (2.347)	< 0.001

^a^Mean (SD); *n* (%).

^b^Wilcoxon rank sum test; Pearson's Chi-squared test.

### Correlation analysis of VI, sleep quality, psychological disorders, and cognitive function

**Figure 2:**
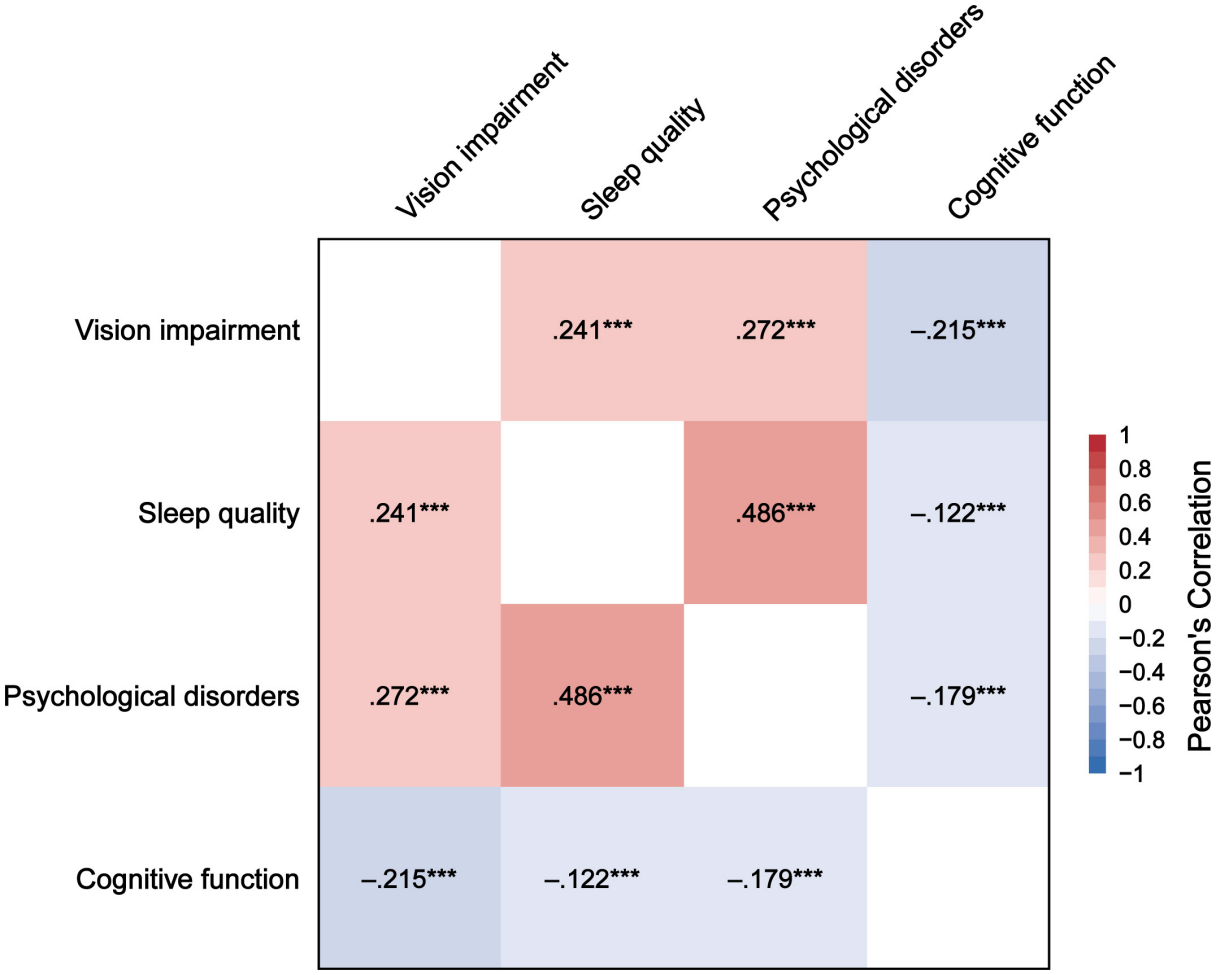
Correlation heatmap of vision impairment, sleep quality, psychological disorders, and cognitive function. ***P < 0.001.

Figure 2 presents the correlations between VI, sleep quality, psychological disorders, and cognitive function. VI is positively correlated with sleep quality (*r* = 0.241, *P* < 0.001) and psychological disorders (*r* = 0.272, *P* < 0.001), and negatively correlated with cognitive function (*r* = −0.215, *P* < 0.001). Sleep quality is positively correlated with psychological disorders (*r* = 0.486, *P* < 0.001), and negatively correlated with cognitive function (*r* = −0.122, *P* < 0.001). Psychological disorders are negatively correlated with cognitive function (*r* = −0.179, *P* < 0.001).

### VI and cognitive function: chain mediation test

In light of the possible connection between sleep quality and psychological disorders, our research constructed two chain mediation models to explore how sleep quality and psychological disorders mediate the correlation between VI and cognitive function (Chain Mediation Model 1: from VI to sleep quality to psychological disorders to cognitive function; Chain Mediation Model 2: from VI to psychological disorders to sleep quality to cognitive function). The analysis utilized the PROCESS function with Model 6 in the R package bruceR (version 2024.6) (36) to examine the chain mediation models. Age, gender, drinking status, residence, marital status, education, total wealth, race, smoking status, BMI, and number of diseases were included as adjustment variables in this research.

**Figure 3:**
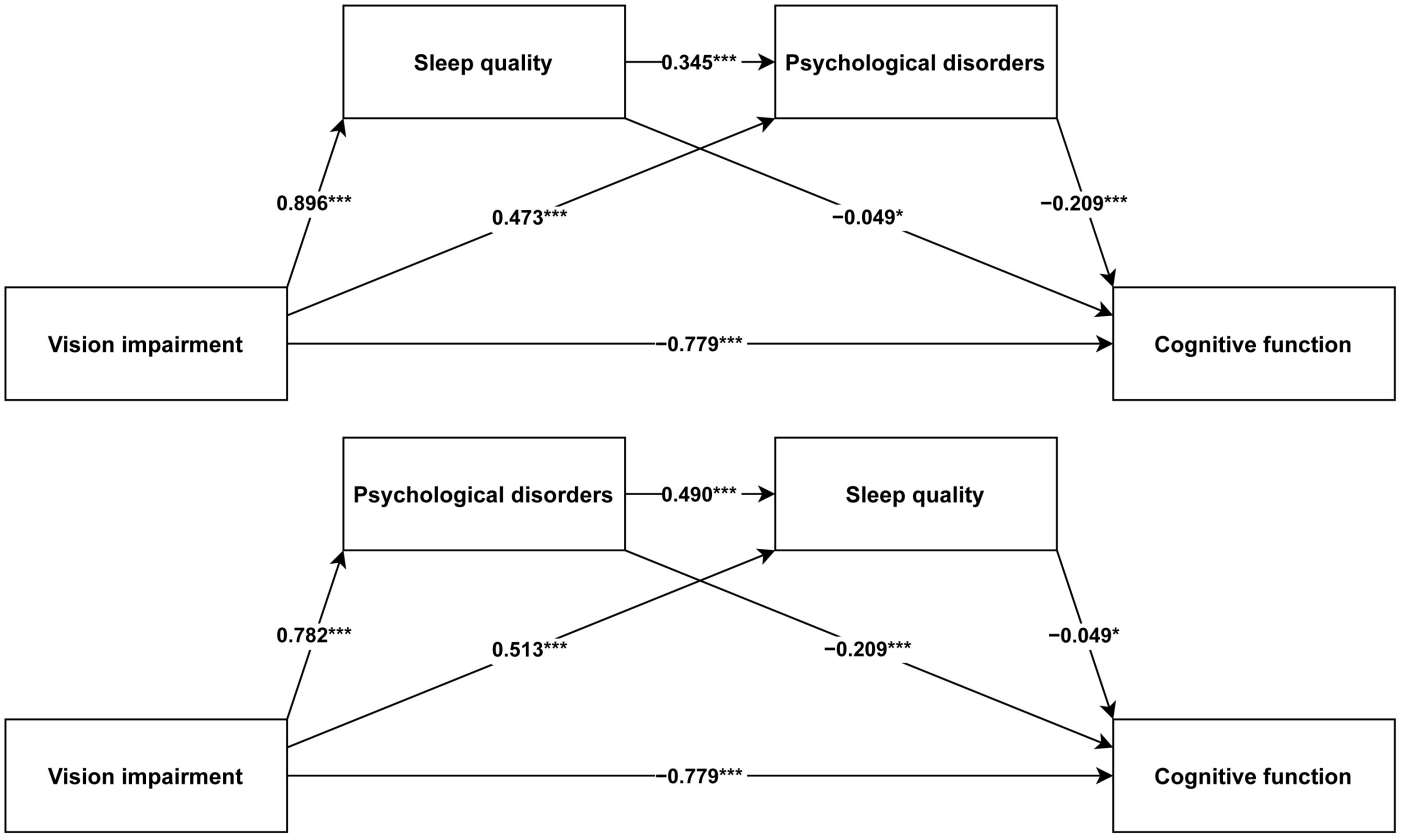
Chain mediation model results. *P < 0.05. ***P < 0.001.

Figure 3 illustrates the path coefficients for both chain mediation models, with all paths being statistically significant. Table 2 presents the regression analysis results of the two chain mediation models. All in all, VI has a negative impact on cognitive function (*BETA* = −0.779, *P* < 0.001). Sleep quality (*BETA* = −0.049, *P* = 0.015) and psychological disorders (*BETA* = −0.209, *P* < 0.001) negatively affect cognitive function. For Chain Mediation Model 1, VI has a positive impact on sleep quality (*BETA* = 0.896, *P* < 0.001) and psychological disorders (*BETA* = 0.473, *P* < 0.001), and sleep quality has a positive impact on psychological disorders (*BETA* = 0.345, *P* < 0.001). For Chain Mediation Model 2, VI has a positive impact on psychological disorders (*BETA* = 0.782, *P* < 0.001) and sleep quality (*BETA* = 0.513, *P* < 0.001), and psychological disorders have a positive impact on sleep quality (*BETA* = 0.490, *P* < 0.001).

**Table 2:** Regression analysis results of chain mediation models.

**Dependent variables**	**Independent variables**	***R* ^2^**	***F***	***BETA***	***SE***	***t***	**95% CI**
**Chain Mediation Model 1**							
Sleep quality	Vision impairment	0.120	71.335	0.896	0.050	19.246^***^	(0.805, 0.987)
Psychological disorders	Vision impairment	0.303	216.093	0.473	0.040	13.050^***^	(0.402, 0.544)
Psychological disorders	Sleep quality	0.303	216.093	0.345	0.010	47.007^***^	(0.331, 0.360)
Cognitive function	Vision impairment	0.261	168.326	−0.779	0.090	−8.541^***^	(−0.957, −0.600)
Cognitive function	Sleep quality	0.261	168.326	−0.049	0.020	−2.431^*^	(−0.088, −0.009)
Cognitive function	Psychological disorders	0.261	168.326	−0.209	0.020	−8.734^***^	(−0.256, −0.162)
**Chain Mediation Model 2**							
Psychological disorders	Vision impairment	0.161	100.686	0.782	0.040	20.011^***^	(0.705, 0.859)
Sleep quality	Vision impairment	0.268	182.379	0.513	0.040	11.870^***^	(0.428, 0.598)
Sleep quality	Psychological disorders	0.268	182.379	0.490	0.010	47.007^***^	(0.469, 0.510)
Cognitive function	Vision impairment	0.261	168.326	−0.779	0.090	−8.541^***^	(−0.957, −0.600)
Cognitive function	Psychological disorders	0.261	168.326	−0.209	0.020	−8.734^***^	(−0.256, −0.162)
Cognitive function	Sleep quality	0.261	168.326	−0.049	0.020	−2.431^*^	(−0.088, −0.009)

^*^P < 0.05.

^***^P < 0.001.

In the two chain mediation models, Table 3 presents the total, direct, and indirect effects associated with different paths. The total effect of VI on cognitive function is −0.986 (−1.169, −0.808). The direct effect of VI on cognitive function is −0.779 (−0.966, −0.598). The indirect pathways of the two models are described as follows:

**Table 3:** Bootstrap analysis of the mediating effects of sleep quality and psychological disorders.

**Model pathways**	***BETA***	***SE***	**95% CI**	**Mediating effect (%)**
**Chain Mediation Model 1**				
**Total effect**				
VI → Cognitive function	−0.986	0.092	(−1.169, −0.808)	100.0
**Direct effect**				
VI → Cognitive function	−0.779	0.094	(−0.966, −0.598)	79.0
**Indirect effects**				
VI → Cognitive function	−0.208	0.023	(−0.254, −0.163)	21.0
VI → Sleep quality → cognitive function	−0.044	0.018	(−0.079, −0.008)	4.4
VI → Psychological disorders → cognitive function	−0.099	0.014	(−0.129, −0.072)	10.0
VI → Sleep quality → psychological disorders → cognitive function	−0.065	0.009	(−0.082, −0.049)	6.6
**Chain Mediation Model 2**				
**Total effect**				
VI → Cognitive function	−0.986	0.092	(−1.169, −0.808)	100.0
**Direct effect**				
VI → Cognitive function	−0.779	0.094	(−0.966, −0.598)	79.0
**Indirect effects**				
VI → Cognitive function	−0.208	0.023	(−0.254, −0.163)	21.0
VI → Psychological disorders → cognitive function	−0.164	0.021	(−0.207, −0.123)	16.6
VI → Sleep quality → cognitive function	−0.025	0.011	(−0.046, −0.005)	2.5
VI → Psychological disorders → sleep quality → cognitive function	−0.019	0.008	(−0.034, −0.003)	1.9

Chain Mediation Model 1

Path 1: VI → Sleep quality → cognitive function, with an indirect effect of −0.044 (−0.079, −0.008), accounting for 4.4% of the total effect.
Path 2: VI → Psychological disorders → cognitive function, with an indirect effect of −0.099 (−0.129, −0.072), accounting for 10.0% of the total effect.
Path 3: VI → Sleep quality → psychological disorders → cognitive function, with an indirect effect of −0.065 (−0.082, −0.049), accounting for 6.6% of the total effect.

Chain Mediation Model 2

Path 1: VI → Psychological disorders → cognitive function, with an indirect effect of −0.164 (−0.207, −0.123), accounting for 16.6% of the total effect.
Path 2: VI → Sleep quality → cognitive function, with an indirect effect of −0.025 (−0.046, −0.005), accounting for 2.5% of the total effect.
Path 3: VI → Psychological disorders → sleep quality → cognitive function, with an indirect effect of −0.019 (−0.034, −0.003), accounting for 1.9% of the total effect.

All indirect effects are statistically significant because none of the bootstrap 95% confidence intervals include the value 0.

## Discussion

The number of individuals with cognitive impairment doubles approximately every 20 years due to the increasing aging population. Caregivers and family members supporting these patients are commonly experiencing heightened levels of stress and diminished psychological wellbeing (5, 37). An inquiry into the causes of cognitive decline is imminent.

This study primarily focused on investigating the relationship between VI and cognitive function and assess the mediating role of psychological disorders and sleep quality. After adjusting for the influences of sleep quality, psychological disorders, and covariates, the study found that VI still had a negative impact on cognitive function, aligning with the findings of previous research (37–39). Recent research has demonstrated that the thinning of the outer retina in patients with age-related macular degeneration (AMD) is related to diminished cognitive performance (40). A case-control study found that individuals suffering from primary open-angle glaucoma (POAG) are more prone to exhibit cognitive impairment compared to age-matched controls (41). A longitudinal study raised speculation that there could be a correlation between VI and potential cognitive dysfunction in the future (42). A notable relationship exists between visual function indices and cognitive performance, as measured by Montreal Cognitive Assessment (MoCA) scores, in individuals with mild cognitive impairment (MCI) (43). The numerous studies mentioned above suggest a potential association between VI and cognitive function. Therefore, timely measures to prevent the onset or alleviate the symptoms of VI may be of great importance in preventing cognitive decline.

The correlation between VI and cognitive function has been widely studied for its important role. However, only a few studies have considered the chain mediation role of other factors in the relationship. We then proposed the hypothesis that there is a chain-mediated effect of sleep quality and psychological disorders between VI and cognitive function and constructed a model of the chain mediation effect in our research.

### Mediation of the association between VI and cognitive function by sleep quality

Results from this study suggest a partial mediation effect of sleep quality on the link between VI and cognitive function. It was observed that VI positively predicted sleep quality, meaning that individuals with VI experienced poorer sleep quality than those who did not have VI. Research has revealed that across all age groups, from children to older adults, individuals with VI typically experience reduced sleep duration and quality compared to their sighted counterparts (44–47). Though the concrete mechanisms linking VI and sleep have not been fully explored, there are already some reasonable theories available. One study suggests that external light is important for managing the body's internal biological clock (48), after being received by the eyes, light triggers signals that pass through several pathways to reach the suprachiasmatic nucleus (SCN), which serves as the central clock synchronizing multiple biological rhythms into a 24-h circadian cycle (49). For individuals with VI, the reduced stimulation of the optic nerve by light input makes it challenging to synchronize their sleep cycle, potentially leading to recurring insomnia and daytime drowsiness (50). Additionally, VI results in decreased exposure to ambient light, which reduces the production of melatonin (51), thereby negatively affecting sleep quality (52). At the same time, people with VI tend to avoid going outside or reduce the frequency of outdoor activities due to mobility difficulties (53). This also further decreases their exposure to bright light. Individuals with VI maintain a prolonged state of alertness to prevent accidents, which leads to increased daily stress, longer sleep latency, and greater susceptibility to sleep disruptions, such as frequent awakenings (48). Our research also suggests that sleep quality is negatively correlated with cognitive function, the poorer the sleep quality, the poorer the cognitive function. Good sleep quality, as a solid guarantee of personal health, has long been the focus of various researchers, and as the study of cognitive function has become a hot topic in recent years, its links with cognitive function have also been gradually uncovered (7, 54, 55). Research shows that lack of quality sleep may exacerbate neurodegeneration through the promotion of neuroinflammation and the disruption of neurogenesis, especially within the hippocampal areas, a vital region for memory and learning (56). Previous studies have indicated that adequate sleep positively impacts cognitive performance through a physiological process increasing the volume of white matter and decreasing the volume of gray matter in the brain. This structural change enhances mental stability and cognitive performance, as demonstrated in experimental findings highlighting the beneficial effects of sufficient sleep on adolescents (7). A recent study used data from the UK Biobank to develop a non-linear model whose longitudinal analysis showed that U-shaped length of sleep showed a connection to cognitive decline (47, 55). Based on these findings, it may be possible to alleviate symptoms of cognitive decline, such as memory loss and loss of verbal organization, in individuals with VI by improving their sleep.

### Psychological disorders mediate the association between VI and cognitive function

Our study shows that VI further influences cognitive function through the mediating role of psychological disorders. According to the results, patients with VI have a heightened susceptibility to psychological disorders. The strong correlation has been validated in the literature. Data analyzed across the region suggests that VI populations are worse off psychologically and have a higher risk of mental illness, which is consistent with our research results (57–62). Regarding how VI impacts mental health, one study posits that VI may pose greater challenges in communication and reduce an individual's motivation for social activities, thereby intensifying the sense of social isolation. Additionally, individuals with VI often experience a reduced quality of life and face additional financial burdens compared to those with normal vision, further exacerbating the stress of their lives (19). Nevertheless, certain research investigating the relationship between VI and depressive symptoms has generated conflicting results. As an illustration, a longitudinal study focusing on older adults in Australia found no substantial link between VI and depressive symptoms (63). This indicates that the intricate relationship between VI and psychological disorders requires further investigation and clarification. Similarly, when psychological health is poor, cognitive function tends to suffer, as evidenced by numerous studies, aligning with our analysis. Among individuals suffering from major depressive disorder, changes in hypothalamic-pituitary-adrenal (HPA) axis activity and heightened cortisol production have been observed (64), while anxiety patients also exhibit variations in cortisol levels (65); a growing body of evidence indicates a connection between increased cortisol and cognitive decline, as well as dementia in older adults (66). Ella Cohn-Schwartz's theory further suggests that mental health problems can negatively impact cognitive function over time by reducing motivation to participate in both real-world and online social activities (67). However, the absence of evidence makes it quite difficult to confirm these hypotheses and refine the relevant theories, leaving deeper connections yet to be uncovered. It is noteworthy that even if visual acuity improves and psychological wellbeing is enhanced in patients with VI following cataract surgery, preoperative anxiety, and other psychological states can still adversely affect mental wellbeing and surgical outcomes (58). Therefore, the combined impact of these factors must be considered in treatments aimed at improving both visual and psychological wellbeing.

### Bidirectional chain mediation of associations between VI and cognitive function by sleep quality and psychological disorders

Our study suggests that sleep quality and psychological disorders act as bidirectional chain mediation between VI and cognitive function and that sleep quality is positively related to psychological disorders (poorer sleep quality is linked to the worsening of psychological statuses) and vice versa. Research indicates that sleep disturbances can be viewed as one expression of circadian rhythmicity disruption (CRD) and that CRD serves as a shared psychopathological factor (p-factor) among most mental health issues (68). Those who have anxiety or related disorders typically report low-quality sleep and are often affected by various sleep disturbances, especially insomnia (69). Major Depressive Disorder (MDD) is a common psychological disorder, with sleep disturbances being one of its primary clinical manifestations. In recent years, numerous studies have revealed the neural substrates underlying the relationship between sleep and depression from a neurobiological perspective. Firstly, compared to MDD patients with normal sleep efficiency (SE), those with lower SE show abnormalities in the structure of brain white matter. For example, alterations in the white matter integrity of the corona radiata and internal capsule have been observed, which may be closely related to their sleep disturbances (70). Secondly, changes in resting-state functional connectivity (rsFC) also influence the relationship between SE and symptoms of anxiety and depression (71). Additionally, a longitudinal study on MDD patients demonstrated that an increase in baseline rapid eye movement (REM) sleep percentage facilitates the transition from an acute depressive state to remission. This effect is mediated by the regulation of brain activity in the left inferior temporal gyrus and cerebral blood flow in the bilateral central lobules (72). Notably, patients with MDD tend to exhibit longer REM latency and shorter REM duration, which are associated with decreased voxel-mirrored homotopic connectivity (VMHC) in the precentral gyrus and inferior parietal lobule (73). Mendelian randomization is a method that utilizes genetic proxies for exposure to assess causal relationships with outcomes of interest. By integrating multiple Mendelian randomization studies, researchers have found a bidirectional causal relationship between neuropsychiatric disorders and sleep-related phenotypes, indicating a close connection and mutual influence between the two (74). Overall, sleep and mental state are recognized as deeply interconnected. It has been established by our study that sleep quality and psychological disorders as bidirectional chain mediators linking VI with cognitive function, which implies that understanding cognitive deterioration in VI patients requires a more expansive viewpoint. When enhancing the cognitive function of VI patients, the synergy between sleep and psychological factors suggests that addressing both aspects simultaneously could yield better results than focusing on just one. Likewise, improving only a single factor might lead to diminished outcomes if the other aspect remains unaddressed or performs poorly. Ultimately, greater assessment accuracy is achieved when sleep and mental state are embedded in the cognitive evaluation of VI patients.

### Limitation

Some limitations of this study could be resolved in future research. First, long-term longitudinal follow-up data is a better choice for studying causal relationships and mediating effects, and the findings of the validation study should be further validated multiple times using longitudinal data. Second, this study focuses solely on the population of older adults in the United States aged 50 and above. Due to the specific regional and age range covered, there are challenges in extrapolating the results to other age groups or regions. Notably, in younger populations, the normal health status and the prevalence of various diseases differ significantly from those in older adults. Moreover, considering that most regions worldwide are characterized by low-resource environments, disparities in access to healthcare services, health awareness, and environmental factors can lead to variations in disease prevalence and manifestation. These differences may influence the strength and pathways of the associations observed in this study. Additionally, racial/ethnic diversity may contribute to variations in the study results. Variations exist among different racial/ethnic groups in disease prevalence, healthcare access, and psychological health services, which could impact the study's conclusions. Although the HRS data include multiple racial and ethnic groups, some subgroups have limited sample sizes, restricting their representativeness and the generalizability of the results. Future research should pay greater attention to different racial/ethnic populations, conducting targeted studies to verify and expand findings across diverse demographic and socioeconomic backgrounds, thereby enhancing the applicability and value of the conclusions. Third, this study primarily relies on self-report data to construct key variables. For VI and sleep quality, objective clinical measurements (such as visual acuity tests, contrast sensitivity assessments, polysomnography, actigraphy, etc.) and validated questionnaire tools (e.g., Pittsburgh Sleep Quality Index, PSQI) were not used. Instead, only subjective assessments by individuals were employed, which may be biased due to differences in personal perception, varying levels of health awareness, and inaccuracies in memory. Fourth, in our study, we did not sufficiently account for additional confounding factors, such as certain individuals having diseases that affect cognitive decline (e.g., thyroid dysfunction, vitamin B_12_ deficiency, vitamin D deficiency, malnutrition, acute illnesses, etc.), but lacking corresponding self-reports. Additionally, some participants were lost to follow-up due to death or other reasons. These individuals tend to have poorer health status, which could potentially lead to an underestimation of the true effect of VI on cognitive function. Fifth, some of the mediating effects identified in this study are relatively small (for example, accounting for only 1.9%−6.6% of the total effect), suggesting that these variables serve only as partial mediators. It is likely that other unmeasured key pathways exist. Therefore, in clinical practice, these findings should be interpreted with caution to avoid overestimating their practical significance. Future research should aim to incorporate multiple mediating variables across different dimensions to comprehensively elucidate the underlying mechanisms. Nonetheless, even small effect sizes can still hold meaningful implications, particularly in large-scale population interventions, where they may contribute to public health impact.

## Conclusion

Findings from this trial cover several areas, including the complex links and interactions between VI, cognitive function, sleep quality, and psychological disorders. The use of sleep quality and psychological disorders as bi-directional mediators suggests that the correlates between VI and cognitive function in the aging populations may be complex and interactive and that cognitive enhancement can be achieved by improving the patient's sleep quality and psychological disorders, or by integrating sleep quality and psychological disorders scales into the assessment of cognitive function as well for comprehensive consideration.

## Data Availability

Publicly available datasets were analyzed in this study. This data can be found here: https://hrs.isr.umich.edu/.
